# Biomarkers Predictive of Response to Thiopurine Therapy in Inflammatory Bowel Disease

**DOI:** 10.3389/fmed.2020.00008

**Published:** 2020-01-29

**Authors:** Jack S. Cornish, Elisa Wirthgen, Jan Däbritz

**Affiliations:** ^1^University Hospital Geelong, Barwon Health, Geelong, VIC, Australia; ^2^Department of Pediatrics, Rostock University Medical Center, Rostock, Germany; ^3^Center for Immunobiology, The Barts and the London School of Medicine and Dentistry, Blizard Institute, Barts Cancer Institute, Queen Mary University, London, United Kingdom

**Keywords:** intestinal inflammation, outcome, predictors, Crohn's disease, ulcerative colitis, thiopurine, azathioprine, 6-mercaptopurine

## Abstract

The complex nature of inflammatory bowel disease (IBD) often results in treatment failure for many patients. With some patients cycling through multiple therapies before achieving a sustained period of remission, the ability to predict a patient's response to therapeutics could decrease the time from active disease to clinical remission and mucosal healing. The prospect of such individualized treatment of IBD would be aided by accurate biomarkers, both fecal and serological, which have to date shown value as indicators of IBD activity. Here we review the utility of generic biomarkers for inflammation or mucosal healing, such as calprotectin, C-reactive protein (CRP), and fecal hemoglobin (fHb) as predictors of response to treatment of IBD. We further provide a deeper insight into the utility of monitoring the thiopurine treatment by thiopurine metabolites or alternative hematologic parameters. In light of multiple recent publications of biomarkers and biological therapy, our focus in this review is predicting response to thiopurine treatment only, that is, Azathioprine and 6-Mercaptopurine.

## Introduction

Inflammatory bowel disease (IBD) is a complex disease with multiple risk factors, interactions and treatment options, in the context of the important clinical need to rapidly establish and maintain mucosal healing (MH) (1). The goal of being able to individualize therapy for patients with IBD, so as to maximize effectiveness—including rapid induction of MH—whilst minimizing side effects, has not progressed as quickly in IBD as in other diseases, such as cancer (2). The ability to predict clinical response before the instigation of therapy, or shortly thereafter, would allow for rapid escalation of therapy when required with minimization of side effects. Currently, Crohn's disease (CD) and ulcerative colitis (UC) have multiple different scoring systems using both clinical factors and biomarkers (usually serum-based) to determine disease severity and likely outcome, but less in terms of predicting treatment response. This review article will focus mainly on biomarkers that are currently in clinical use. To date much of the research surrounding response prediction in IBD is centered around treatment with biological agents, such as Infliximab (IFX) (3–10). Given recent reviews published elsewhere in this area (11, 12), our review will only cover studies related to predicting a response to immunomodulator treatment with either azathioprine or 6-mercaptopurine since this was not covered by the before mentioned review articles.

## Methodology

The review was performed using PubMed/MEDLINE up to June 2019. The search strategy was using the following search terms alone or in combination: monitoring, biomarker, marker, surrogate, evaluation, prediction, predictor, response, responder, healing, recurrence, relapse, remission, management, efficacy, outcome, flare, immunomodulators, immunosuppressants, thiopurine, azathioprine, 6-mercaptopurine, methotrexate, therapy, treatment, induction, maintenance, serum, fecal, fecal, blood, lactoferrin, S100, C-reactive protein, serological, microbiome, intestinal flora, microflora, gut flora, interleukin, mucosal, mucosa, polymorphisms, inflammatory bowel disease, Crohn's disease, ulcerative colitis. Boolean operators (“not,” “and,” “or”) were also used in succession to narrow or widen the search. The primary outcome was to review the clinical utility of biomarkers for the prediction of response to treatment with immunomodulators in IBD.

## Thiopurine Therapy

The thiopurine metabolites, azathioprine (AZA) and 6-mercaptopurine (6-MP), have a long history of use as immunomodulators in patients with IBD (13). Whilst initially having been used based on their known effectiveness in other autoimmune inflammatory diseases, there is now a strong evidence for their use to maintain remission in IBD (13–16). Furthermore, as shown in the SONIC trial, combination therapy of oral AZA with IFX is seen to be superior over monotherapy with either agent for inducing steroid free remission in CD (17).

## Thiopurine Metabolism and Modes of Action

**Figure 1:**
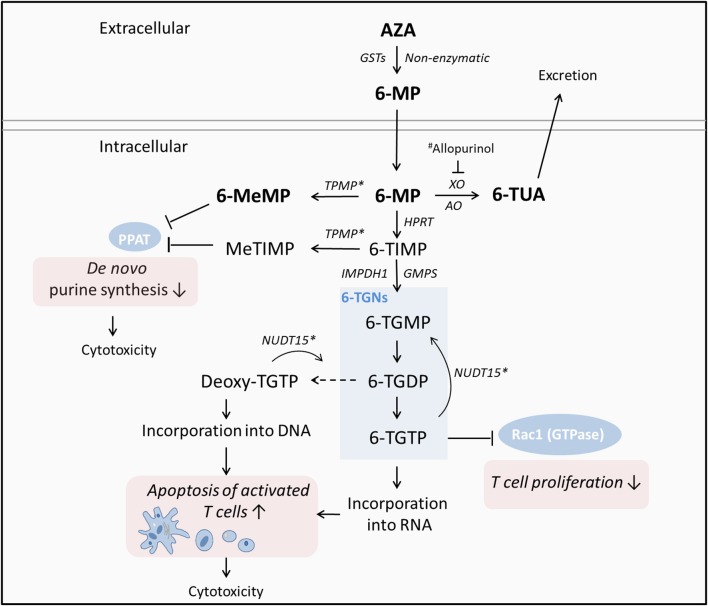
Simplified metabolism of thiopurines and modes of action according to (19–21). As a prodrug, AZA is converted to 6-MP upon reaching the systemic circulation. Following the uptake by transporter molecules, 6-MP is metabolized by three competing pathways either in the liver or gut resulting in immunosuppressive effects. Importantly, 6-TGNs serve as the active metabolites of thiopurine therapy, incorporating into lymphocyte DNA and thereby inducing apoptosis of activated T-lymphocytes as well as exerting direct cytotoxic effects at higher doses. In addition, 6-TGTP inhibits the activity of the GTPase Rac1 resulting in suppression of T cell-dependent immune response. The thiopurine metabolites 6-MeMP and MeTIMP inhibit the enzyme PPAT which catalyzes the first step of *de novo* purine synthesis; resulting in inhibition of DNA synthesis and cell proliferation along with cytotoxic effects. AZA, Azathioprine; 6-MP, 6-mercaptopurine; TPMT, thiopurine S-methyltransferase; TUA, thiouric acid; HPRT, hypoxanthine phosphoribosyltransferase; MeMP, methylmercaptopurine; TIMP, thioinosine monophosphate; TGNs, thioguanine nucleotides; XO, xanthine oxidase; AO, aldehyde oxidase; TGMP, guanosine monophosphate; TGDP, guanosine diphosphate; TGTP, guanosine triphosphate, NUDT15, nudix hydrolase 15; PPAT, phosphoribosyl pyrophosphate aminotransferase; Rac1, Rac family small GTPase 1. ^*^Associated with variability in tolerance to thiopurines. ^#^XO inhibitor allopurinol, applied to induce a switch toward 6-TGN production in patients who do not adequately respond to thiopurine treatment.

As a prodrug, AZA is converted to 6-MP by glutathione transferases (GSTs) or non-enzymatically upon reaching the systemic blood circulation. Following the intestinal uptake by transporter molecules, 6-MP is metabolized by three competing pathways either in the liver or gut (18, 19) resulting in immunosuppressive effects (Figure 1). Importantly, 6-thioguanine nucleotides (6-TGNs) serve as the active metabolites of thiopurine therapy, incorporating into lymphocyte DNA and thereby inducing apoptosis of activated T-lymphocytes (22) as well as exerting direct cytotoxic effects at higher oncologic doses (23) (Figure 1). In addition, 6-TGN thioguanosine triphosphate (TGTP) inhibits the activity of GTPase Rac1 resulting in suppression of T cell-dependent immune response (22, 24). The conversion of 6-MP into 6-methylmercaptopurine (6-MeMP) or methylthioinosine monophosphate (meTIMP) by thiopurine methyltransferase (TPMT), inhibits the enzyme phosphoribosyl pyrophosphate (PPAT) which catalyzes the first step of *de novo* purine synthesis (25). As a consequence, there is inhibition of DNA synthesis and cell proliferation along with cytotoxic effects (26).

Genetic polymorphisms affecting the activity of specific enzymes in the thiopurine pathway are associated with adverse drug reactions due to a shift of metabolite distribution (19). Patients with a complete or partial deficiency of TPMT, where one or two gene copies are defective, have a high risk of developing severe myelosuppression during treatment with standard doses with AZA or 6-MP (27). In Caucasians, ~10% of the population are heterozygous for a TPMT allele causing TPMT deficiency while only 0.3–0.5% are homozygous (20). Due to the preferred metabolism to 6-TGNs, these patients are more responsive to thiopurines where lower dosages are necessary. Thus, patients with complete TPMT deficiency should avoid thiopurine treatment or, if necessary, start with <10% of standard dosage; whereas individuals with a heterozygous genotype should be treated with 50% of standard initiation dose (28). In contrast to TPMT deficiency, the overexpression of TPMTs is associated with an increased accumulation of MeMP, with concurrent lower levels of 6-TGNs. This is referred to as thiopurine hypermethylation and is associated with drug toxicity and non-response to thiopurine treatment (29). In this context it has been shown that the co-therapy with allopurinol, an inhibitor of the enzyme xanthine oxidase, can improve the production of 6-TGNs by modifying 6-MP metabolism (30) promoting clinical remission and mucosal healing (31–34). Regarding the proven relevance of TMPT activity for the outcome of thiopurine treatment, measuring TMPT levels prior to starting of therapy is recommended in Caucasians to prevent potentially life-threatening myelotoxicity (35, 36). In this context, phenotyping using an enzyme assay, is preferred to genotyping, given completely deficient patients can be detected in any case irrespective of the TPMT variant (37). In contrast, the prevalence of non-wild type alleles in the Asian population is much lower, with <5% of population being heterozygous and almost none being completely deficient.

In addition to TMPT, variants of the gene NUDT15 affects the metabolism of thiopurines (38). The enzyme NUDT15 dephosphorylates the active thiopurine metabolites TGTP and deoxyTGTP, thus preventing their incorporation into DNA (39). It is assumed that decreased NUDT15 enzymatic activity or a lower expression level is associated with a higher level of active 6-TGNs resulting in thiopurine-induced myelotoxicity and a reduced thiopurine maintenance dose (38, 40). In addition to toxic effects on bone marrow function, AZA and 6-MP are associated with a high risk of hepatotoxicity (41) with an incidence of 1 in 133 in a population based study from Iceland (42). Although the prognosis is generally favorable, patients with pre-existing cirrhosis should be considered carefully before starting a thiopurine therapy (43).

## Monitoring of Thiopurine Treatment

To evaluate the efficacy and potential toxicity of thiopurine treatment, the concentration of thiopurine metabolites can be assessed in red blood cells (RBC). However, there are some analytical pitfalls aggravating the translation of metabolite levels to clinical outcome (44). Although it has been shown, that lower TGN concentrations were associated with the development of active Crohn's disease, there was substantial variation between repeated measurements of 6-TGNs on the same patient (45) revealing that serial 6-TGN analyses are required to evaluate active metabolite levels. Furthermore, due to high costs or limited availability of the analytical tests, the therapeutic monitoring of TGNs is not practical for every clinic (46). Therefore, surrogate markers, such as the white blood cell count (WBC) or the mean corpuscular volume (MCV) are discussed as alternatives in monitoring thiopurine efficacy.

### Thiopurine Metabolites

With regard to the metabolism of thiopurines, it is assumed that very high or very low levels of metabolites are associated with adverse reactions or non-response to thiopurine treatment. As a consequence, 6-TGNs and 6-MMP are often evaluated to gauge the success of thiopurine therapy. In spite of some studies identifying a strong association between metabolite levels and clinical outcome, the current viewpoint is not yet clear enough for routine clinical use of metabolite levels as predictors of treatment response (Table 1).

**Table 1:** Studies assessing the value of biomarkers in predicting response to thiopurine treatment in IBD.

**Study**	**Patient population**	**Subjects (n)**	**Therapy**	**Metabolite/Biomarker**	**Cut off level**	**Hazard Ratio** **Odds Ratio or *P*-value**	**Sensitivity/Specificity (%)**	**PPV/NPV (%)**	**Definition of relapse**	**Definition of response**
Park et al. (47)	CD Pediatric/Adult	82	AZA/6-MP	CRP	≥5 mg/L	*p* = 0.009	–	–	CDAI > 150	CDAI < 150 (remission)
Lémann et al. (48)	CD Adult	83	AZA	CRP Hb	≥20 mg/L <12 g/dL	*p* = 0.0002 *p* = 0.043	–	–	CDAI > 250 OR CDAI between 150 and 250 on 3 consecutive weeks with an increase of at least 75 points above baseline OR the need for surgery for CD (with the exception of limited perianal surgery)	–
Treton et al. (49)	CD Adult	66	AZA	CRP Hb NC	≥20 mg/L <12 g/dl >4 × 10^9^/l	HR 58.6, *p* = 0.002 HR 4.8, *p* = 0.04 HR 3.2, *p* = 0.003	–	–	HBI ≥ 4 with the need for treatment	HBI ≤ 3 without any steroid treatment or immunosuppressive agent in the past 3 months and without surgery.
Osterman et al. (50)	CD/UC Adult	971	AZA/6-MP	6-TGN	≥230 pmol/8 × 10^8^ RBCs	OR = 3.27	62/67	–	Not provided	Not provided
Nguyen et al. (51)	CD/UC Pediatric	86	AZA	6-TGN	≥250 pmol/8 × 10^8^ RBCs	OR = 4.4 *p* = 0.007	–	–	Inability to achieve steroid free remission	PCDAI ≤ 10 without corticosteroids PUCAI ≤ 10 without corticosteroids
Kwan et al. (52)	CD/UC Adult	39	AZA/6-MP	6-TGN	≥230 pmol/8 × 10^8^ RBCs	–	63/67	63/67	Disease flare requiring cyclosporine, infliximab, intravenous corticosteroid	Any reduction in oral steroid use, HBI, SCCAI scores, and clinical assessment
Gonzalez-Lama et al. (53)^a^	CD/UC Adult	113	AZA/6-MP	6-TGN	≥230 pmol/8 × 10^8^ RBCs	–	41/56 at 2 weeks 58/50 at 1 month 57/50 at 2 months 68/62 at 4 months	61/36 at 2 weeks 64/44 at 1 month 67/40 at 2 months 84/40 at 4 months	Unable to achieve steroid free CDAI <150	CDAI <150 or mTWAI <11 without corticosteroids for at least 6 months
Gonzalez-Lama et al. (53)^b^	CD/UC Adult	113	AZA/6-MP	6-TGN	260 pmol/8 × 10^8^ RBCs	–	35/65 at 2 weeks 49/53 at 1 month 53/57 at 2 months 57/75 at 4 months	63/37 2 weeks 62/40 1 month 68/41 2 months 87/37 4 months	Unable to achieve steroid free CDAI <150	CDAI <150 or mTWAI <11 without corticosteroids for at least 6 months
Waljee et al. (54)	CD/UC Adult	346	AZA/6-MP	6-TGN	–	AuROC = 0.594	–	–	mHBI ≥ 4 on or off steroids or; mHBI <4 requiring steroids	CD—mHBI <4, steroid free, no fistulae for 3 weeks UC—mUCDAI <4 off steroids
Nakarai et al. (55)	UC Adult	158	AZA/6-MP Biologics	fHb	<100 ng/mL	–	92/71	37/97	–	MS = 0
Mooiweer et al. (56)	CD/UC Adult	164	AZA/6-MP Biologics	fHb	1.5 μg/g	–	74/84	72/84	–	Assessed ability to predict MS
Takashima et al. (57)	UC Pediatric/Adult	98	AZA/6MP Biologics	FIT fCP	<100 ng/mL <250 μg/g	–	95/62 82/62	–	–	Assessed ability to predict MES

*AuROC, Area under Receiver Operator Characteristic Curve; AZA, Azathioprine; CD, Crohn's Disease; CDAI, Crohn's Disease Activity Index; CRP, C-Reactive Protein; fCP, fecal calprotectin; fHb, fecal Hemoglobin; FIT, fecal immunochemical tests; Hb, hemoglobin; HBI, Harvey-Bradshaw Index; mHBI, modified Harvey-Bradshaw Index; MES, Mayo endoscopic subscore; MS, Mayo Score; mTWAI, modified Truelove-Witts Activity Index; mUCDAI, modified Ulcerative Colitis Disease Activity Index; NC, neutrophil count; PUCAI, Pediatric Ulcerative Colitis Activity Index; PPV/NPV; negative/positive predictive value; RBCs, Red Blood Cells; SCCAI, Simple Clinical Colitis Activity Index; UC, Ulcerative Colitis; 6-MP, 6-Mercaptopurine; 6-TGN, 6-Thioguanine nucleotides. Gonzalez-Lama et al. (53) tested two different cut-off levels in the same study indicated by the superscript letters a and b*.

In 2006, Osterman et al. (50) discussed the results of their meta-analysis of 55 retrospective and cross sectional studies focused on 6-TGN levels and remission. In spite of wide variance in each cohort demographic, results were consistent, providing a cut-off of 230–260 pmol/8 × 10^8^ erythrocytes as a therapeutic target in patients with active disease. Above this level, the authors found patients to be 3.3 times more likely to be in clinical remission. Response prediction had a 62% sensitivity and 72% specificity in this study. However, no optimal time point was suggested for testing 6-TGN levels.

Two years following this review, Kwan et al. (52) published the results of their retrospective analysis of a small cohort (*n* = 39) of adults receiving either 6-MP or AZA for treatment of IBD. Although mean 6-TGN values were higher in responders than in non-responders a statistical significance was not achieved (*p* = 0.37). Nevertheless, there was a trend for a better clinical response in patients with 6-TGN levels >230 pmol/8 × 10^8^ RBC, however, the results were statistically insignificant and had a relatively poor accuracy (63% sensitivity, 67% specificity). These results may in part be influenced by the fact that some patients received concomitant corticosteroid and the cohort included the thiopurine treatments 6-MP, AZA, and combined 6-MP/AZA, however, results were collectively analyzed as a whole. Interestingly, in this study, the combination of thiopurine methyltransferase (TPMT) activity (<30.5 U) with 6-TGN metabolite levels (>230 pmol/8 × 10^8^ RBC) was the best predictor of response to thiopurine treatment (*p* = 0.01). Gonzalez-Lama et al. (53) provide important data in their prospective study of 113 adult IBD patients starting immunosuppression with either 6-MP or AZA given a history of steroid refractory luminal disease. Importantly, 6-TGN levels were taken at 2 weeks and then again at 1, 2, and 4 months after commencing thiopurine therapy. No clinically useful cut off could be identified at any time that would allow 6-TGN levels to predict their primary outcome of 6 months steroid free remission. Sensitivity and specificity values were overall very poor (41% sensitivity and 56% specificity at 2 weeks), but did increase as treatment progressed (68% sensitivity and 62% specificity at 4 months). In being the first study that aims to assess the role of 6-TGN levels early on in therapy as markers of long-term treatment response, further larger studies are required to provide more useful data for translation into clinical practice.

A larger 2010 retrospective study of 346 IBD patients from Waljee et al. (54) compared the predictive accuracy of thiopurine metabolites compared to common laboratory markers. Two hundred and eighteen patients diagnosed with CD were included in the study, where the authors developed an algorithm of laboratory values and patient demographics, comparing results to thiopurine metabolite levels in both responders and non-responders. Such laboratory values included neutrophil count, C-reactive protein (CRP), mean corpuscular volume, alkaline phosphatase and thrombocyte count among others. The areas under the receiver operator characteristics curve (AuROC) of the algorithm using laboratory values and patient age was 0.856 (95%CI 0.793–0.919) and was statistically significantly more predictive of treatment response to thiopurines (*p* = 0.001) than the measurement of 6-TGN [AuROC = 0.594 (95%CI 0.546–0.642)]. However, as acknowledge by the authors, the patients in the study were perhaps more likely to have thiopurine refractory disease given that recruitment was from a pool of patients at a single tertiary care center where referral of complex patients is common. Further validation of such a model in a prospective analysis is required prior to treatment being adjusted accordingly.

In 2013, Nguyen et al. (51) retrospectively analyzed 86 pediatric IBD patients whose 6-TGN and 6-MeMP levels were taken at 2 monthly intervals during a median of 18 months of AZA therapy. Their analysis of 6-TGN levels was in accordance with the review article provided by Osterman et al. (50), having attained a statistically significant cut off of 250 pmol/8 × 10^8^ erythrocytes at which disease remission was 4.4 times as likely (*p* = 0.007). Again however, no accurate time point for the cut off was suggested. When evaluating the role of 6-MMP, Nguyen et al. (51) found it was not able to predict response to AZA.

The studies discussed above are an example of both the heterogeneity of populations analyzed and the differing results published when it comes to thiopurine metabolite use as a novel biomarker for predicting response to immunomodulator therapy in IBD.

Yet, in what is perhaps the most comprehensive meta-analysis of 6-TGN levels and remission in IBD, Estevinho et al. (58) systematically reviewed all published evidence of thiopurine use in IBD up to 2017. After screening over 1,000 studies identified by searching four online databases, 25 studies were ultimately deemed eligible for analysis of 6-TGN mean value and cut off levels. Naturally, there was marked heterogeneity in study design and size analyzed, yet mean 6-TGN levels were seen to be higher in those in clinical remission across the board, with a pooled difference of 63 pmol/8 × 10^8^ RBC. A global analysis of cut of levels used found that patients with a 6-TGN level above cut off were 3.95 times more likely to be in remission. Additionally, when the thresholds were analyzed separately, the highest odds ratio (4.71) was found when using a cut off of 250 pmol/8 × 10^8^ RBC.

With these results being similar to the aforementioned 2006 meta-analysis from Osterman et al. (50), the current evidence surrounding the relationship between 6-TGN levels and clinical remission may be used to guide clinical decisions. However, the ability of 6-TGN levels to accurately predict response to therapy with thiopurines is currently clinically insignificant given the modest sensitivity and specificity values published to date.

### Blood Cell Counts

As a simple and low-cost alternative to measure concentrations of 6-TGNs in patients during AZA/6-MP treatment, the evaluation of hematologic parameters, such as counts of white or red blood cells are discussed here as surrogate markers.

Studies in 166 patients with inflammatory bowel disease reveal that the mean corpuscular volume (MCV) correlated with the RBC 6-TGN concentration (*r* = 0.33, *p* < 0.001) (59). This was supported by a 5-years database study (60) revealing a positive correlation between MCV and 6-TGN. Furthermore, it has been shown that the change of MCV (ΔMCV) correlates with intracellular 6-TGN levels which may be useful for monitoring of intracellular metabolite levels (46). In contrast to MCV, RBC count, WBC and absolute neutrophil count (ANC) correlated negatively with RBC 6-TGN concentrations during AZA or 6-MP treatment (*r* = −0.16, −0.28, −0.19, respectively). Interestingly, the concentration of the thiopurine metabolite 6-MeMP did not correlate with the specified hematologic parameters. Although in patients, who received a 6-TGN treatment the WBC and the platelet count correlated positively with the 6-TGN concentration, this was, however, not reproducible in different disease subgroups indicating the effect of 6-TGNs alone on bone marrow function is rather slight compared to a combination of different downstream metabolites of AZA or 6-MP.

According to the assumption that 6-TGN levels correlate with the efficacy of thiopurine therapy it was evaluated whether hematologic surrogate markers for thiopurine metabolites are suitable for prediction of remission or relapse. In this context, a meta-analysis in 2002 included 424 patients with IBD revealed that the ANC, WBC as well as the MCV were predictive factors of achieving remission after 6 months of AZA treatment (61). Therefore, the ANC and the WBC were higher in patients which achieved remission while the MCV was lower compared with patients where remission was not achieved (*p* = 0.0001). Furthermore, Cox regression analysis revealed that in patients who achieved remission a WBC < 5.0 × 10^9^ was associated with a lower risk of relapse (*p* = 0.03). In the SONIC trial, an ΔMCV > 7 after 26 weeks of oral AZA therapy was associated with a higher proportion of steroid-free remission (62). However, the analysis of 15 studies did not provide sufficient evidence that ΔMCV can predict clinical remission after AZA or 6-MP treatment (46).

## Biomarkers of IBD Activity

Induction and maintenance of clinical remission, characterized by the absence of mucosal damage and inflammation is one main focus of IBD treatment (63). In this context, hematologic biomarkers, non-specific to IBD or treatment, such as CRP are commonly used to monitor states of inflammation. Additionally, fecal biomarkers, such as fecal calprotectin (fCP), fecal immunochemical tests (FIT) or fecal hemoglobin (fHb) are discussed as biomarkers for mucosal healing.

### Hematological Biomarkers

CRP is an acute phase protein primarily synthesized in the liver and released from hepatocytes following proinflammatory signaling via interleukin (IL-) 1 and IL-6 along with tumor necrosis factor-alpha (TNF-α) (7, 8). In a non-inflammatory state, CRP levels are often <1 mg/L with rapid increases in concentration observed following acute inflammatory events (64). As such, CRP is a long-known serum marker for inflammation within the body and is currently used for diagnostic purposes and as a means of measuring disease activity in IBD. However, the high titres of CRP often observed in CD are not common in UC patients, where it is common to see little to no elevation in CRP concentration (5, 65–67). With CRP concentrations shown to fluctuate with disease activity, results regarding its utility in predicting relapse are to date variable (Table 1). Furthermore, due to genetic polymorphisms in the CRP gene, 20–25% of CD patients do not produce increased CRP levels during acute inflammation (68). In these patients CRP levels would not reflect internal states of inflammation and are therefore not suitable as a surrogate marker of (intestinal) inflammation.

Whilst there is limited literature solely focused on CRP predicting response to thiopurine treatment, a 2012 study from Park et al. (47) retrospectively observed 82 CD patients who received their first course of AZA or 6-MP with initial fortnightly follow up for the first month before commencing three monthly reviews. Aged between 14 and 45 years, patients with a CRP >5 mg/L at the time of remission induced by thiopurine treatment were more likely to experience relapse than those with a CRP <5 mg/L (*p* = 0.009). The vast majority of patients with an elevated CRP experienced relapse within 24 months of remission induction.

From a different stand point, Lémann et al. published in 2005 their results of observing 83 adult CD patients over the first 18 months after withdrawal of AZA or 6-MP treatment (48). Whilst the primary outcome of their study was to identify any difference in the time to relapse between patients who continued AZA or received a placebo after an initial period of 2 years on AZA, the authors noted that a CRP level >20 mg/L as well as a Hb level <12 g/dL after 2 years of AZA treatment, did in fact predict relapse to occur within the next 18 months of treatment when it came to sub-analysis.

Additionally, Treton et al. (49) followed the Lemann et al. (48) cohort further to assess response to AZA treatment in patients in clinical remission after continuous treatment with AZA for at least 42 months. After AZA interruption the median follow-up period were 47 months. Univariate analysis identified that a CRP level >20 mg/L after AZA interruption predicted relapse (*p* = 0.002). Independently from CRP, the blood levels of Hb < 12 g/dl and NC > 4 × 10^9^/l were associated with an increased risk of relapse (*p* = 0.04, *p* = 0.003, respectively).

Whilst we discuss the role of biomarkers for prediction of relapse elsewhere (63), these results support a high CRP following long term AZA treatment as being predictive of treatment failure.

### Fecal Biomarkers

As an indicator of underlying mucosal damage, gastrointestinal blood loss is an important feature of IBD and is beginning to gather interest as a future biomarker of disease activity. In spite of its use as a screening tool in colorectal cancer (69), the utility of fecal immunochemical tests (FITs) as a marker of occult blood loss in IBD has been studied to a far lesser extent. In ulcerative colitis patients, FIT and calprotectin effectively reflect the state of mucosal inflammation and detect active UC better than remission (70). As a result, FITs may be useful in reducing the need for invasive endoscopic examination. The degree of research focused on fHb as a biomarker of intestinal inflammation is itself limited, with only a handful of studies assessing its utility in comparison with FC. McDonald et al. showed low fHb to be an accurate rule out study for IBD activity when comparing to colonoscopy (71). Similar finding were shown elsewhere where negative fHb was concluded as a good indicator of no significant bowel disease (72). Considering the fact that fHb is a general marker for mucosal healing and not specific for thiopurine therapy, we provide a brief account of studies where a large proportion of participants received thiopurine therapy and identify fHb as a biomarker able to predict disease activity with accuracy comparative to that of the well-studied fCP (Table 1). In these studies, all data was analyzed as a collective irrespective of treatment, as such the accuracy in which results predict response to thiopurines is confounded by participants receiving other therapeutic agents.

In 2012, Nakarai et al. (55) published their findings having evaluated the relationship between colonoscopy findings and FIT result in 158 adult patients with UC. With 42% of these patients treated with thiopurines, fecal samples were taken on the day of colonoscopy or within a month of the colonoscopy for FIT. The authors found a fHb level <100 ng/mL predicted mucosal healing as defined by a Mayo score (73) of 0 or 1 at colonoscopy with a sensitivity of 60% and specificity of 87%. Sensitivity increased to 92% when predicting a Mayo score of 0 at the same cut off with a reduction in specificity to 71%. When using a cut off of <60 ng/mL the predictive value was not any more superior for Mayo score 0 or 1 with a modest increase when predicting a Mayo score of 0. Two further studies have been published comparing the predictive value of FIT to fCP in adult CD and UC patient populations. The first of which compared the two biomarkers against the level of intestinal inflammation found during surveillance colonoscopies (56). Forty percent of the 164 patients received thiopurine therapy, 63% of all patients had a Mayo score of 0. The ability to predict inflammation in both UC and CD patients was seen to be comparable between both fCP and FIT. Using a cut-off of 1.51 μg/g, fHb predicted inflammation with 74% sensitivity and 84% specificity relative to the 86% sensitivity and 72% specificity provided by a fCP level above 140 mg/g. Pertinent to our review, the authors did not provide data regarding the long-term treatment response in these patients. Similar results were found by Takashima et al. (57) in 2015, however they found the FIT values to be slightly more sensitive (95%) in predicting a Mayo score of 0 at a level <100 ng/ml compared to multiple fCP cut-offs (77% <200 μg/g and 82% at 250 μg/g). Furthermore, detailed reviews on the broader role of FIT in IBD compared to fCP (74) described a similar school of thought where an increase in sensitivity seen between FIT compared to fCP is often noted when predicting the more strict Mayo score of 0. Given the large body of evidence surrounding fCP and IBD more broadly, the current comparative accuracy of FIT makes it promising low-cost way of measuring disease activity and with more tailored future investigation, a candidate biomarker for predicting treatment response upon the publication of such studies in the future.

Calprotectin is a calcium-binding protein complex of two damage-associated molecular pattern proteins (DAMP), S100A8/S100A9, mainly found in neutrophils (75, 76). It can be measured in feces by enzyme-linked immunosorbent assay (ELISA), and is known to correlate well with intestinal inflammation (75). Its use in distinguishing between irritable bowel syndrome and IBD is well-established, and it is being increasingly used to inform management of those with established IBD (77). Its potential use for the prediction of treatment responses is a new area of investigation for the use of calprotectin (Table 1). The stated cut-off values of fCP for clinical significance reported in many studies is not standardized, and they are often quoted in individual studies at levels that optimize the sensitivity and specificity seen in each individual cohort. In clinical practice, given not only the wide variety of presentation and severity of disease, but also the known variability of intra-individual fCP levels with active UC (78, 79), a percentage decrease from baseline fCP level might be more informative. With regard to the role of fCP in predicting response to thiopurine therapy, there are to our knowledge to date no published studies that interpret its predictive utility. The detailed role of fCP in IBD along with its ability to predict response to other forms of treatment than thiopurines alone are described in a number of up to date reviews (11, 12, 63, 80–84).

## Conclusions

**Figure 2:**
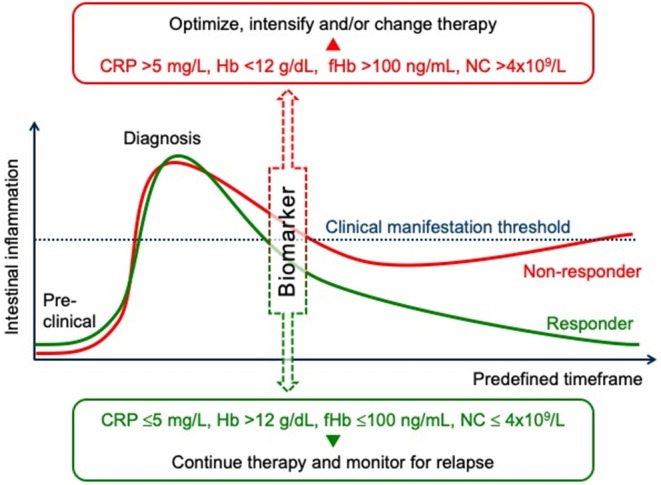
Prediction of treatment responses in inflammatory bowel diseases. An outline of disease activity, from preclinical symptoms through to remission as indicated by a clinical manifestation threshold. At a predefined stage of the induction therapy (e.g., after 1–3 months according to the treat to target strategy), biomarkers, such as C-reactive protein (CRP), hemoglobin (Hb), fecal hemoglobin (fHb), or neutrophile count (NC) correlate with intestinal inflammation and can predict the response to treatment. Early assessment of treatment efficacy using such surrogate markers, and in conjunction with other biochemical tests, clinical signs, and/or imaging studies, can help to adjust treatment in case of persistent inflammatory disease activity. Such an individualized approach/algorithm can help to achieve mucosal healing and thus to avoid long-term bowel damage and subsequent disability.

Currently, the role of biomarkers in predicting treatment response to thiopurine therapy is not as well-studied compared to the large body of current evidence surrounding the role of biomarkers in biological therapies (11, 12, 63, 85). The current understanding of fCP in particular as not only a marker of disease activity but also as predictor of relapse and treatment response is well-studied in the context of biological therapy but not yet in patients treated with immunomodulator monotherapy. Fecal CP has been clearly shown to correlate with intestinal inflammation. With strong evidence for its use as a surrogate marker for intestinal inflammation (77) we have previously discussed the increasing evidence for its use in predicting relapse (63). Although more evidence is required, it appears that fCP is the most promising of all biomarkers studied to date in the context of individualizing IBD treatment (Figure 2). However, its role in predicting thiopurine response is poorly studied. Unfortunately, whilst many studies assessing the predictive value of fCP include patients on thiopurines, there is no sub-analyses of these cohorts alone and as such there is no real knowledge of how it represents the efficacy of immunomodulation. To see future studies assessing the role of fCP as predictor of thiopurine response would be important not only for guiding thiopurine treatment, but also to provide more robust knowledge of the potential use of fCP in all IBD patients.

Similarly, the role of CRP is to date studied in a small number of modest sized cohorts with CD. Its ability to predict response to thiopurine treatment is encouraging based upon on the findings of our review and even more so given the wider evidence base for its role in guiding IBD treatment.

The measurement of thiopurine metabolite levels is currently the most well-studied potential biomarker for predicting response to thiopurine treatment. Yet, the level of evidence is still well below what would be required for a safe translation into clinical practice. A greater number of large prospective trials and randomization is yet required prior to 6-TGN and 6-MeMP levels being used to individualize IBD treatment.

## Future Directions

The focus of this article has been predominantly in terms of investigations that are already currently in clinical use. However, development of basic science knowledge, particularly in genomics and immunology, does suggest to us what clinicians will use in the future. The reader is directed to some excellent reviews on the use of genetics and immunology in predicting treatment responses and disease course (1), including the use of gene expression signatures from involved tissue and genetic variability at non-IBD susceptibility loci, for example, apoptosis-related genes for T cells (86, 87). The early detection of treatment failure and prediction of treatment responses are important in maximizing efficiency, and minimizing cost and side effects, and can be considered to be the first step in achieving the goal of individualized IBD management (88). The low population response rate of most treatment regimens for IBD lends itself to the emerging paradigm of CD and UC having many subtypes (89), therefore, increasing the importance of tailoring treatment to the individual. It is likely that the four described “-omes” that together determine the development of IBD and its clinical course (exposome, microbiome, immunome, and genome) (90) may well also affect treatment response (1). Therefore, as we gain further insights into each of these domains our ability to predict treatment outcome will improve. Given the many treatment options for IBD which are approaching on the horizon further research in the prediction and accurate determination of treatment response will be ever more critical.

## Author Contributions

JC and JD analyzed and interpreted the data. JC wrote the first draft of the article. JD and EW created the figures. JD and EW contributed to the writing of the article. All authors performed the literature review, read and approved the final version of the manuscript.

### Conflict of Interest

The authors declare that the research was conducted in the absence of any commercial or financial relationships that could be construed as a potential conflict of interest.

## Abbreviations

6-MP: 6-mercaptopurine
6-TGNs: 6-thioguanine nucleotides
6-MeMP: 6-methylmercaptopurine
AuROC: area under the receiver operator characteristics curve
ANC: absolute neutrophil count
AZA: azathioprine
CRP: C-reactive protein
CD: Crohn's disease
CDAI: Crohn's Disease Activity Index
DNA: deoxyribonucleic acid
GTP: Guanosin triphosphate
GTPase Rac1: Rac family small GTPase 1
fCP: fecal calprotectin
fHb: fecal hemoglobin
FIT: fecal immunochemical tests
Hb: hemoglobin
HBI: Harvey-Bradshaw Index
IBD: inflammatory bowel disease
IFX: infliximab
IL: interleukin
meTIMP: methylthioinosine monophosphate
MCV: mean corpuscular volume
MH: mucosal healing
mHBI: Modified Harvey-Bradshaw Index
MS: Mayo score
mTWAI: Modified Truelove-Witts Activity Index
mUCDAI: Modified Ulcerative Colitis Disease Activity Index
NC: neutrophil count
NPV: negative predictive value
NUDT15: nudix hydrolase 15
PPAT: phosphoribosyl pyrophosphate
PPV: positive predictive value
PUCAI: Pediatric Ulcerative Colitis Activity Index
RBC: red blood cells
SCCAI: Simple Clinical Colitis Activity Index
TGTP: thioguanosine triphosphate
TPMT: thiopurine methyltransferase
UC: ulcerative colitis
WBC: white blood cell count.
